# Predicting Biologic Therapy Outcome of Patients With Spondyloarthritis: Joint Models for Longitudinal and Survival Analysis

**DOI:** 10.2196/26823

**Published:** 2021-07-30

**Authors:** Carolina Barata, Ana Maria Rodrigues, Helena Canhão, Susana Vinga, Alexandra M Carvalho

**Affiliations:** 1 Instituto Superior Técnico Universidade de Lisboa Lisbon Portugal; 2 Instituto de Telecomunicações Instituto Superior Técnico Universidade de Lisboa Lisbon Portugal; 3 Comprehensive Health Research Center NOVA Medical School NOVA University of Lisbon Lisbon Portugal; 4 EpiDoC Unit, The Chronic Diseases Research Centre NOVA Medical School NOVA University of Lisbon Lisbon Portugal; 5 Instituto de Engenharia de Sistemas e Computadores: Investigação e Desenvolvimento em Lisboa (INESC-ID) Instituto Superior Técnico Universidade de Lisboa Lisbon Portugal; 6 Lisbon Unit for Learning and Intelligent Systems Lisbon Portugal

**Keywords:** data mining, survival analysis, joint models, spondyloarthritis, drug survival, rheumatic disease, electronic medical records, medical records

## Abstract

**Background:**

Rheumatic diseases are one of the most common chronic diseases worldwide. Among them, spondyloarthritis (SpA) is a group of highly debilitating diseases, with an early onset age, which significantly impacts patients’ quality of life, health care systems, and society in general. Recent treatment options consist of using biologic therapies, and establishing the most beneficial option according to the patients’ characteristics is a challenge that needs to be overcome. Meanwhile, the emerging availability of electronic medical records has made necessary the development of methods that can extract insightful information while handling all the challenges of dealing with complex, real-world data.

**Objective:**

The aim of this study was to achieve a better understanding of SpA patients’ therapy responses and identify the predictors that affect them, thereby enabling the prognosis of therapy success or failure.

**Methods:**

A data mining approach based on joint models for the survival analysis of the biologic therapy failure is proposed, which considers the information of both baseline and time-varying variables extracted from the electronic medical records of SpA patients from the database, Reuma.pt.

**Results:**

Our results show that being a male, starting biologic therapy at an older age, having a larger time interval between disease start and initiation of the first biologic drug, and being human leukocyte antigen (HLA)–B27 positive are indicators of a good prognosis for the biological drug survival; meanwhile, having disease onset or biologic therapy initiation occur in more recent years, a larger number of education years, and higher values of C-reactive protein or Bath Ankylosing Spondylitis Functional Index (BASFI) at baseline are all predictors of a greater risk of failure of the first biologic therapy.

**Conclusions:**

Among this Portuguese subpopulation of SpA patients, those who were male, HLA-B27 positive, and with a later biologic therapy starting date or a larger time interval between disease start and initiation of the first biologic therapy showed longer therapy adherence. Joint models proved to be a valuable tool for the analysis of electronic medical records in the field of rheumatic diseases and may allow for the identification of potential predictors of biologic therapy failure.

## Introduction

### Motivation

Rheumatic diseases are chronic diseases that, being the leading cause of disability in developed countries, consume many health and social resources. Among these diseases, spondyloarthritis (SpA) is a group of several related disorders that can be highly debilitating and significantly impact patients' quality of life, health care systems, and society [1].

As there is no cure, treatment focuses on the relief of symptoms and the delay of the disease's progression. Biologic therapies are the most recent approach for treating these disorders, and their use is recommended when all other methods have failed. However, the therapy selection follows no specific criteria, and trying to establish which patients benefit the most from each drug is still a problem that needs to be solved [2].

A better understanding of therapy responses for these patients and identifying the predictors that affect these responses would allow for a prognosis of therapy success or failure and thus be highly valuable in conserving the resources and time of both patients and medical doctors. Moreover, this understanding could be used to aid medical experts in tailoring the treatment to the patient by using a more personalized approach.

Meanwhile, the emerging availability of electronic medical records has enabled the storage of great amounts of information that can be used to extract insightful knowledge. Data mining is a rapidly growing field that focuses on developing the techniques necessary for insightfully using this information.

The analysis of an outcome of interest is usually performed using survival analysis methods. such as the Kaplan-Meier estimator [3] and the Cox model [4]. Nevertheless, these methods are only able to deal with time-static variables. For dealing with time-varying variables, methods such as the extended Cox model [5] have been introduced. However, they are not appropriate for dealing with biomarkers [6,7].

Joint models have been presented in the literature as a useful approach for handling these types of analysis, having been used in a wide range of medical studies, including the most common disease areas of cancer and HIV and AIDS [8].

In the field of rheumatic diseases, these joint modeling and machine learning approaches were studied to evaluate the clinical impact on flare occurrence in patients undergoing biologic treatments for rheumatoid arthritis. Both models were proven to assist in decisions on biologic dose reduction with the potential to reduce the occurrence of flares significantly [9]. The development of juvenile dermatomyositis was also studied using longitudinal approaches, allowing for a better perception of longitudinal outcomes and a more accurate comprehension of predictors' effects [10].

However, the use of these methods has been less explored for other diseases in this field, such as SpA.

Our main goal was to propose a data mining approach based on joint models to infer relationships between time-to-event and longitudinal electronic medical record data, retrieved from the Rheumatic Disease Portuguese Register (Reuma.pt) [11]. We further aimed to study the predictors of failure of the first biologic therapy for patients with SpA and verify the applicability of joint models for the study of therapeutic response in rheumatic diseases.

### Background

#### Spondyloarthritis

Spondyloarthritis is the name given to a family of inflammatory rheumatic diseases that share distinctive pathophysiologic, clinical, radiographic, and genetic features. This includes ankylosing spondylitis (AS)—the characteristic type of this group—psoriatic arthritis, reactive arthritis, enteropathic arthritis, and so called undifferentiated SpA.

AS is characterized by chronic inflammation predominantly affecting the axial skeleton. Although its pathogenesis is poorly understood, there is a strong association between AS and the human leukocyte antigen B27 (HLA-B27), and the typical age at onset of this condition is at the second or third decade of life [12].

The first population-based study on rheumatic diseases in Portugal, EpiReumaPt, reported the national health survey results in 2015, revealing a general SpA prevalence of 1.6% and a prevalence of 2.0% and 1.2% for women and men, respectively [1].

The socioeconomic impact can be rather high for these conditions. A recent study [13] revealed that AS has a total annual economic impact of €639 million (US $773 million) in Portugal. This value includes the disease-related costs for the patient and the national health system and the economic impact of the lost workdays.

Clinical monitoring of a disease is of extreme importance to understanding disease progression, better assessing patient response to treatment, and guiding therapeutic decisions.

Laboratory exams include erythrocyte sedimentation rate (ESR) and levels of C-reactive protein (CRP), which are markers of inflammation, and other laboratory data that are considered to show relevant alterations.

Functional ability can be evaluated using the Bath Ankylosing Spondylitis Functional Index (BASFI) [14] score, and activity disease can be evaluated using the Bath Ankylosing Disease Status in Ankylosing Spondylitis (BASDAI) score [15] or the more recently developed Ankylosing Spondylitis Disease Activity Score (ASDAS) [16]. The ASDAS has two different formulas, ASDAS-CRP (which uses the C-reactive protein) and ASDAS-ESR (which uses ESR), with ASDAS-CRP usually being the preferred system.

Treatment of SpA should be tailored to the patient, and patient signs, symptoms, and characteristics should be taken into account, with the most common goal being the attainment of a state of inactive disease.

Treatment options can include physical therapy, nonsteroidal anti-inflammatory drugs, disease-modifying antirheumatic drugs, and, if patients remain in a high disease activity state when trying the referred options, treatment with biologic agents, namely tumor necrosis factor inhibitors and interleukin-17 or interleukin-23 inhibitors.

#### Time-to-Event Analysis

Survival analysis, or time-to-event analysis, is the collection of statistical procedures for the analysis in which the outcome variable of interest is the time until an event occurs.

Let *T* denote a random, nonnegative, continuous variable representing the patient’s survival time and let *t* be an observed value of *T*.

The hazard function can be interpreted as the instantaneous potential per unit time for the event to occur, given that the individual has survived to time *t*. It is calculated as follows:


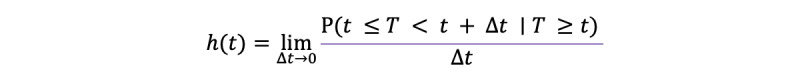


The main feature that distinguishes survival data from other types of data is the possible presence of censored survival times.

Considering a specific individual *i*, let *T_i_* be the random variable representing its true survival time and *C_i_* the potential censoring time. With consideration to right censoring, the censoring indicator variable, *δ_i_*, is defined as *δ_i_* = *I*(*T_i_* ≤ *C_i_*), where *I* (. ) is the indicator function.

The Cox proportional hazards model [4] allows us to estimate the hazard function and explore how the survival of a group of patients depends on the values of one or more explanatory variables.

Let ***x*** = (*x*_1_, *x*_2_, … , *x*_k_) be the values of the *k* explanatory variables of an individual and ***β*** = (*β*_1_, *β*_2_, …, *β_k_*) the vector of its correspondent unknown regression coefficients. The hazard function is given by the following:

*h*(*t*; ***x***) = *h*_0_ (*t*)exp (***β***T***x***)

where *h*_0_ (*t*) is the baseline hazard function, representing the hazard for a patient when its vector of explanatory variables is equal to zero (***x*** = 0).

It is possible to extend the previously presented Cox model for handling time-dependent variables [5]. This model is referred to as the extended Cox model. However, it is not theoretically appropriate to deal with biomarkers since it assumes that the time-dependent variables are predictable processes, measured without error and with a full path completely known.

#### Longitudinal Analysis

Longitudinal data can be defined as the data obtained from multiple measurements of individuals throughout time.

Linear mixed effects (LME) models are a common way of modeling this data. In an LME model, the individual’s response is assumed to follow a linear regression model where some of the regression parameters are population specific and others are patient specific. These are referred to as fixed effects and random effects, respectively [17].

Let **Y***_i_* be the *n_i_*-dimensional response vector for subject *i*. In general, a linear mixed-effects model satisfies the following:


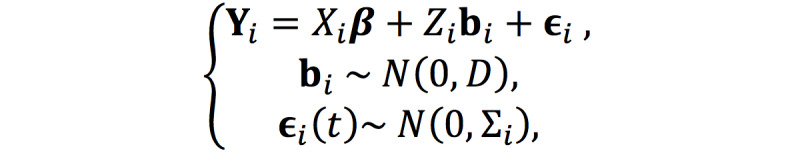


where ***β*** is a *p*-dimensional vector that contains the fixed effects; **b***_i_* is the *q*-dimensional vector containing the random effects, and **ϵ***_i_* is a *n_i_*-dimensional vector of random errors; *X_i_* and *Z_i_* are the (*n_i_* × *p*) and (*n_i_* × *q*) fixed-effects and random effects design matrices, respectively; *D* is a (*q* × *q*) positive-definite covariance matrix; and Σ*_i_* is a (*n_i_* × *n_i_*) positive-definite covariance matrix that depends on *i* through its dimension *n_i_*_._ The **ε***_i_* is normally distributed with mean zero and covariance matrix *D*, and **b***_i_* is normally distributed with mean zero and covariance matrix Σ*_i_*. Both **b***_i_* and **ε***_i_* are assumed to be independent of each other and of groups [17].

#### Joint Models for Longitudinal and Time-to-Event Data

The basic idea of joint models is to perform combined analysis, in which a relative risk model is estimated for the time-to-event outcome, taking into account the effect of the longitudinal data measurements—this is usually done by combining a survival model with a mixed-effects model [6].

The first step is modeling the continuous longitudinal outcomes with LME models. Let **y***_k_* denote the (*nk_i_* × 1) longitudinal response vector for the *k*-th outcome (*k* = 1, … , *K*) and the *i*-th subject that is composed by elements *y_kil_*,which represent the value of the *k*-th longitudinal outcome taken at time point *t_kil_*. Let **b***_ki_* be a vector of random effects and ***β****_k_* a vector of fixed effects. We have that the conditional expectation of **y***_k_* given **b***_ki_*, *η_ki_* (*t*) is modeled through the LME model as follows:


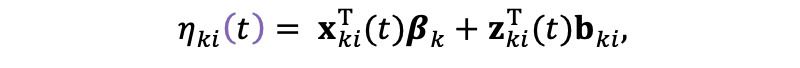


where **x***_ki_* (*t*) and **z***_ki_* (*t*) are the design vectors for the random and fixed effects, respectively.

Let 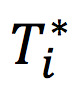 be the true event time for the *i*-th subject. We can now postulate the relative risk model for the survival process as follows:


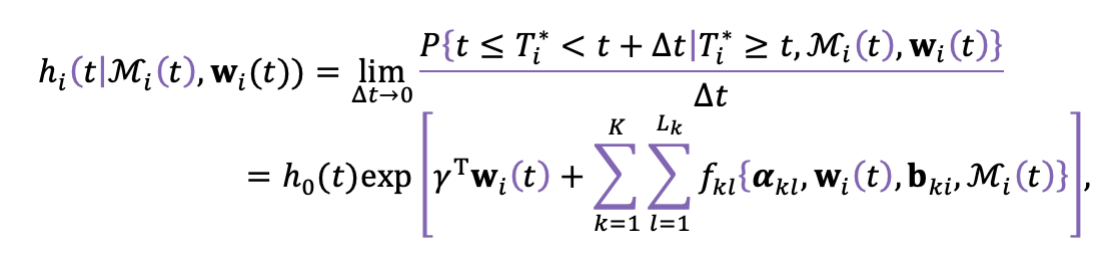


where *M*_i_ (*t*) = {*M*_1_*_i_* (*t*), …, *M_ki_* (*t*)} and *M_ki_*(*t*) = {*η_ki_* (*s*), 0 ≤ *s* < *t*} denotes the history of the true unobserved longitudinal process up to time point *t*, *h*_0_(. ) denotes the baseline risk function, and **w***_i_*(t) is a vector of exogenous covariates with a corresponding vector of regression coefficients γ. The *f_kl_* functions, parametrized by vector ***α****_kl_*, specify which components of each longitudinal outcome will be present in the relative risk model, allowing up to *L*_k_ functional forms for each of *K* longitudinal outcomes. The parameters contained in ***α****_kl_* quantify the effect of the correspondent underlying longitudinal outcome to the risk for an event.

One of the basic approaches for the functional form is to model the event's hazard as having an association only with the current value of the longitudinal outcome at the same time point. Considering a single outcome, this is given by *f*{*α*, **w***_i_*(t), **b***_i_*, *M_i_*(*t*)} = *αη_i_*(*t*), where *α* is the strength of association parameter that indicates the change in the log hazard when there is a unit change in the patient’s longitudinal outcome value.

Estimation of joint models is performed by exploiting the full joint likelihood that is derived from the joint distribution of the longitudinal and survival outcomes. Methods for this estimation can follow, among others, a frequentist or a Bayesian paradigm [18,19].

## Methods

### SpA Patients on Biologic Therapies: Data Description and Preprocessing

The data used in this study were retrieved from Reuma.pt [11] on July 22, 2019. This register was developed by the Portuguese Society of Rheumatology, has been active since June 2008, and contains information retrieved on a routine basis of rheumatic patients in Portugal receiving biological therapies. Although Reuma.pt also contains patients with several rheumatic diseases, the focus of this work was on patients with SpA.

With the data extracted from this database, 4 different data sets, A, B, C and D, were obtained according to different strategies for handling the missing values. A set of different joint models was then fitted to all data sets, as well as the equivalent extended Cox models using R software (The R Foundation for Statistical Computing) [20], namely packages “JM” [18] and “Jmbayes” [19]. This resulted in a total of 49 joint models and 29 extended Cox models. All the steps of the data processing and modeling framework are described in detail in this section.

The Reuma.pt database contains information regarding patients and patient visits, including identification data, demographic data, previous medical history, comorbidities, laboratory results, past and current therapies, adverse events, and disease activity scores, among others.

The follow-up of patients through this registry enables the monitoring of treatment efficacy, safety, and comorbidities.

The goal of this work was to perform a survival analysis that takes into account both time-independent and time-dependent variables and understand how these impact the outcome of interest. Therefore, 3 types of variables were needed: time-independent (baseline) variables, time-dependent variables, and time-to-event variables. The latter type is not directly found in the database and therefore needed to be processed from the existing data.

Our event of interest was the failure of the first biological therapy for each patient, where failure was defined as the discontinuation of the biological therapy due to inefficacy (evaluated by ASDAS) or adverse events (such as infection or hypersensitivity).

In this context, the time-to-event variables indicated if the biologic therapy failed or if the patient was censored—henceforth referred to as the failure index. The time until the occurrence of either failure index is referred to as time to failure.

The data extracted from the database had to go through several preprocessing steps in order to reach a format compatible with the models to be fitted.

First, a set of variables to be considered was selected, with 3 main aspects being taken into account: level of missing values, relevance to the study, and variable equivalence. Variables with more than 60% of values missing were not considered nor were variables that were considered to be irrelevant for the goal of our study as determined according to the feedback given by medical specialists. Furthermore, some variables available in the raw data set were equivalent in the sense that they represented the same information.

After this set of assumptions and processing steps, we obtained our initial data set, which consisted of the following 3 variables:

1. Time-to-event variables—time to failure and failure index;
2. Time-independent variables—sex, marital status, year of diagnosis, age at diagnosis, year of disease beginning, age at disease beginning, year of start of the first biologic therapy, age at start of the first biologic therapy, years from diagnosis to start of the first biologic therapy, disease years until start of the first biologic therapy, HLA-B27, employment status before disease, employment status at baseline, years of education, smoking habits, alcohol consumption habits, weight, height, BMI, number of pathologies, biologic therapy, concomitant disease-modifying antirheumatic drug at baseline, concomitant corticoid at baseline, baseline CRP, baseline ESR, baseline BASDAI, baseline BASFI, and baseline ASDAS;
3. Time-dependent variables—CRP, ESR, BASDAI, BASFI, and ASDAS.

The ASDAS we refer to here and henceforth is the one that incorporates the CR*P* value into its calculation and corresponds to the ASDAS-CRP.

A thorough inspection of all variables was made to identify any incomplete or incorrect values. Some examples of issues that arose were values with incorrect formats or incoherent with these variables’ possible range. If possible, by crossing information and with medical professionals’ help and consultation, the values were corrected, but whenever it was impossible to draw conclusions, the observations were eliminated.

In the presented initial data set, not all patients have every baseline variable available. This poses an issue and is a challenge that needs to be dealt with in most studies that use data from clinical settings. The issue arises because most methods of variable selection and statistical models cannot handle missing values. Therefore, to further proceed with our analysis, we needed to understand the different approaches that can be used to handle this problem according to our needs. Common approaches include performing complete-case analysis, removing individuals with incomplete data for a subset of covariates, and multiple imputation techniques.

We decided not to perform any imputation techniques, as the imputation of baseline variables could introduce a high bias in our models’ estimates.

On the other hand, there is always value in keeping the most amount of data as possible to avoid wasting relevant information.

**Figure 1:**
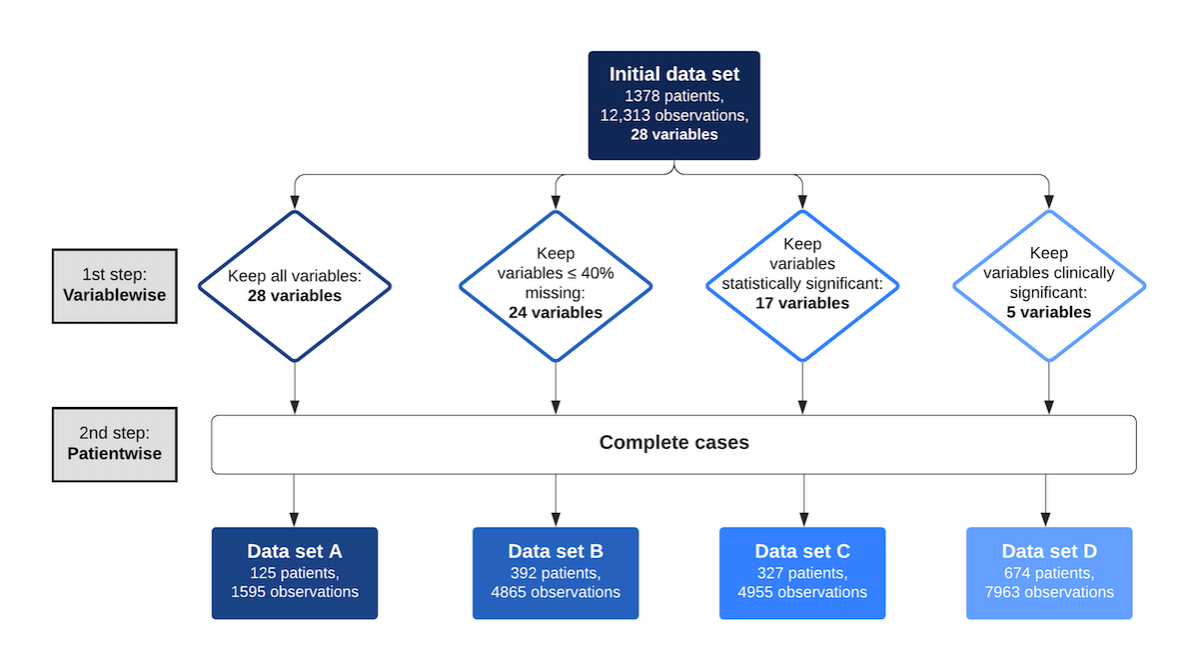
Flowchart representing the overall approach for the preprocessing of the initial data into 4 new data sets: A, B, C, and D.

As there was no obvious choice regarding which approach would be the most appropriate and to enable the drawing of valuable insights from the data, the decision to create 4 different data sets, according to different approaches, was made. Furthermore, this allowed us to study how the strategy for handling missing data and the resulting data differences can influence the modeling process and the subsequent results. The overall process for the creation of these data sets is depicted in Figure 1.

The first approach consisted of keeping only the patients for whom all baseline variables were available (ie, keeping only the complete cases).

The second approach was to consider only the variables with fewer than 40% of missing values and then keeping the complete cases of those variables. The percentage of 40% was chosen since it seemed to provide a good balance between the number of eliminated variables and the number of eliminated patients.

The third approach consisted of fitting a univariate Cox model for each initial baseline variable and then keeping only the statistically significant ones in those models,\ according to a 5% level. After that, the complete cases of those variables were once again kept.

The last approach was to keep only those variables that were considered clinically relevant by expert medical doctors’ knowledge and according to insight from literature research where predictors of biologic drug survival in SpA were studied [21-23].

The variables selected for consideration were sex, disease years to first biologic therapy, age at start of the first biologic therapy, education years, baseline CRP, baseline BASDAI, and baseline BASFI. Variables age at start of the first biologic therapy and baseline BASDAI were later dropped due to violation of the proportional hazards assumption.

### Statistical Model Implementation

For the initial data set, both an overall survival curve and curves for survival according to the biologic therapy were fitted with the Kaplan-Meier estimator [3].

Regarding the 4 processed data sets, the same approach was used for all data sets, which proceeded as follows.

The first step in the analysis was to perform variable selection for the baseline covariates. This removed any unnecessary predictors that could have added noise to the estimations.

Five different methods were used to compare and study the variability of the obtained results. These were backward stepwise selection using Akaike information criterion (AIC), forward stepwise selection using AIC, best subset selection using a primal-dual active set approach, lasso regression, and the stepwise likelihood ratio variable selection strategy presented by Collett [24].

Despite this, the variables obtained from the stepwise likelihood ratio variable selection were the ones ultimately selected for the next steps of the analysis, namely for building the survival submodel.

This variable selection was not performed for data set D, as the medical experts selected the variables of interest for this specific case.

A Cox model for the survival submodel was then fitted using the selected baseline covariates, constituting the survival submodel.

For each of the time-dependent variables, 7 different LME models were fitted. The one with the better fit according to AIC and Bayesian information criterion was chosen as the time-dependent submodel.

The formulae of the different LME models fitted and the corresponding names are presented in Table 1.

Having both submodels, the same joint models were fitted with R packages “JM” [18] and “Jmbayes” [19]. The former estimates the model under a maximum likelihood approach and the latter under a Bayesian approach, more specifically, using Markov chain Monte Carlo algorithms.

The R package “Jmbayes” also enables the fitting of multivariate joint models. Considering that variables CPR and ESR are both measurements of inflammation and that ASDAS uses CRP and BASDAI elements in its composition, we chose not to fit together CRP with ESR or ASDAS with CRP and BASDAI. Thus, 2 different combinations of time-dependent variables were considered for the multivariate joint models: CRP, BASDAI, and BASFI; and BASFI and ASDAS.

The equivalent models, both univariate and multivariate, were also fitted with an extended Cox model, which enabled the comparison of both methods.

Should a case arise where the survival submodel contained any of the variables of baseline CRP, baseline ESR, baseline BASDAI, baseline BASFI, or baseline ASDAS, these baseline variables would be dropped when the correspondent time-dependent variable was present in the univariate joint model or extended Cox model. This would enable us to compare the effect of the variable in its baseline form with its time-dependent form. Similarly, if more than one of the 5 baseline variables were present in the survival submodel, a multivariate joint model or extended Cox model would also be fitted with those variables in the time-dependent form, and the baseline form would be dropped from the survival submodel.

**Figure 2:**
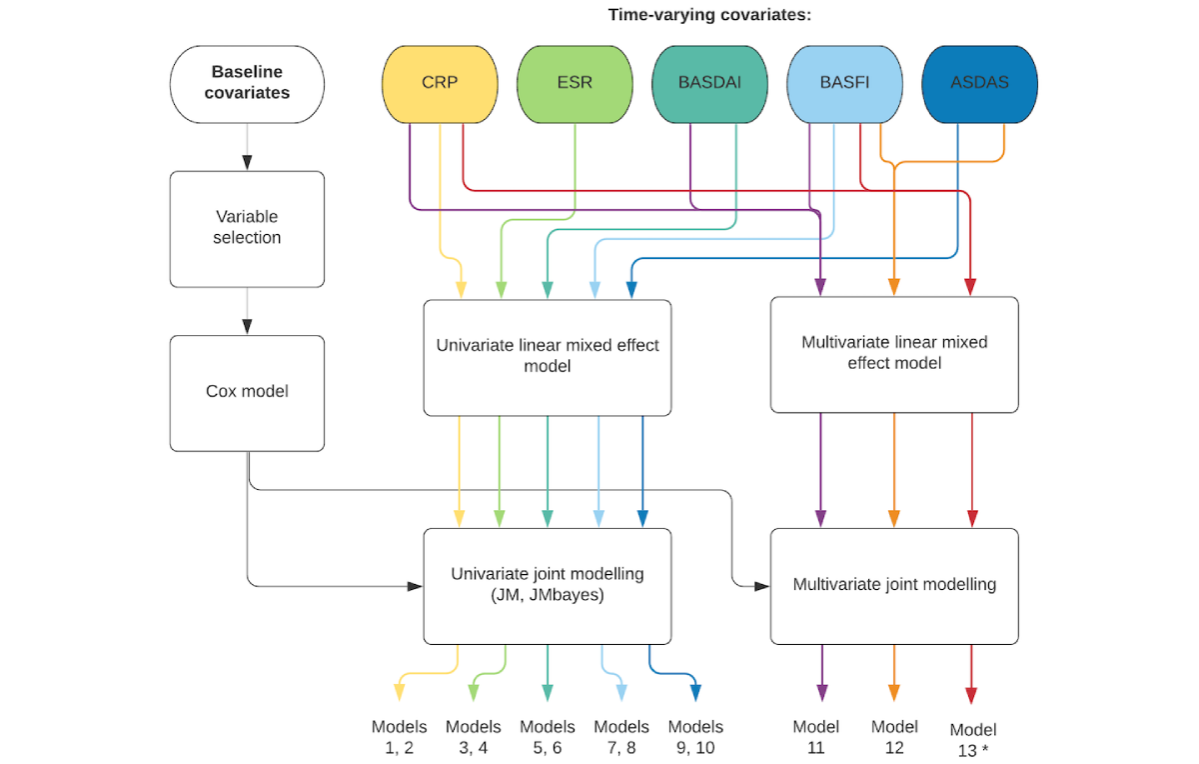
Flowchart representing the overall approach for the data analysis using univariate and multivariate joint modeling. The variable selection step is not performed for data set D. ASDAS: Ankylosing Spondylitis Disease Activity Score; BASDAI: Bath Ankylosing Spondylitis Disease Activity Index; BASFI: Bath Ankylosing Spondylitis Functional Index; CRP: C-reactive protein; ESR: erythrocyte sedimentation rate. *This model is only fitted for data set D.

**Figure 3:**
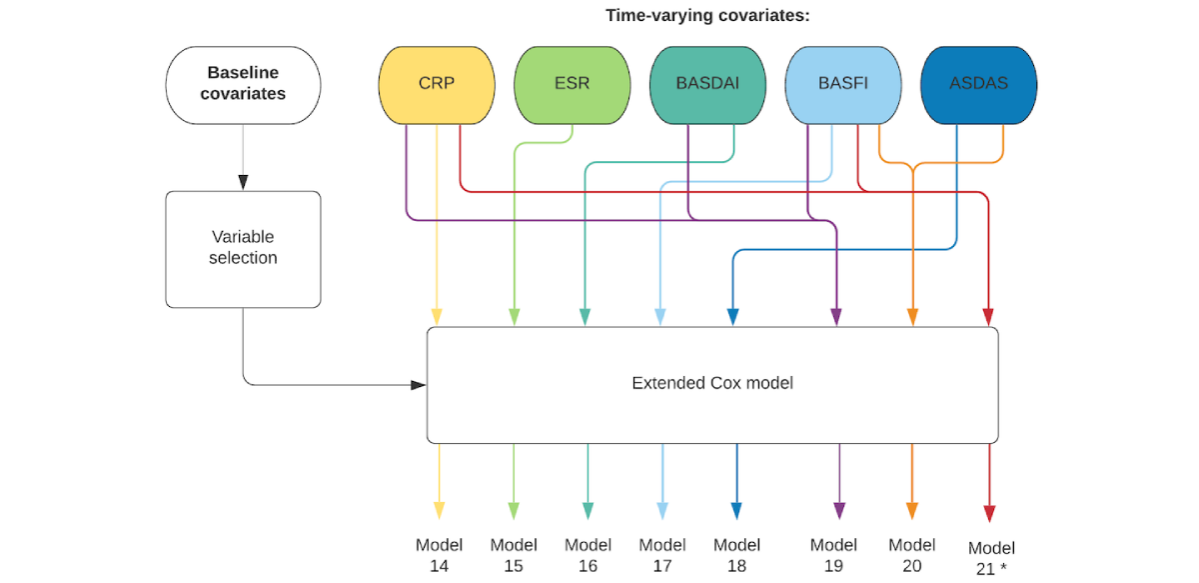
Flowchart representing the overall approach for the data analysis using univariate and multivariate extended Cox modeling. The variable selection step is not performed for data set D. ASDAS: Ankylosing Spondylitis Disease Activity Score; BASDAI: Bath Ankylosing Spondylitis Disease Activity Index; BASFI: Bath Ankylosing Spondylitis Functional Index; CRP: C-reactive protein; ESR: erythrocyte sedimentation rate. *This model is only fitted for data set D.

The overall process for fitting the joint models and extended Cox models is schematically presented in Figure 2 and Figure 3, respectively, where it is also possible to observe the numbers given to the models that were fitted for every data set.

The exhaustive tests performed attempted to cover several types of strategies and models, and were aimed at identifying key covariates involved in the prognosis of the disease, particularly in the response to treatment. Indeed, the rationale for this approach was to comprehensively span the described methods due to the fact that this specific Reuma.pt data set did not contain any prior studies that focused on the identification of specific markers for the prognosis of the patient’s therapy response.

All the analysis was performed using R software [20], particularly, the “MASS” [25], “BeSS” [26], and “glmnet” [27] packages for the forward and backward stepwise variable selection, best subset selection, and lasso regression, respectively. Furthermore “car” [28] was used for multicollinearity testing; “survival” [29] for the Kaplan-Meier curves, Cox model, extended Cox model, and proportional hazards testing; “survminer” [30] for the plotting of survival curves; “nlme” [31] for fitting the linear mixed-effects models; and “JM” [18] and “Jmbayes” [19] for fitting the joint models.

**Table 1:** Time-dependent functions used given a time-varying variable y. NC represents natural cubic spline function; β, fixed effects, b, random effects; and t, time.

Model	Time-dependent functions
Linear and random intercept	*β*_0_ + *β*_1_t_ij_ + *b*_i__0_ + *ε*_ij_
Linear and random slope	*β*_0_ + *β*_1_t_ij_ + *b*_i__0_ + *b*_i__0_*t*_ij_ + *ε*_ij_
Cubic and random intercept	*β*_0_ + *β*_1_t_ij__+_ 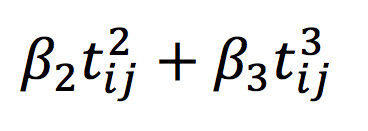 + *b*_i__0_ + *ε*_ij_
Cubic and random slope	*β*_0_ + *β*_1_t_ij__+_ 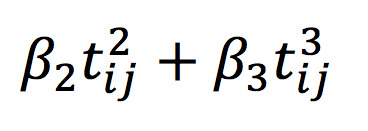 + *b*_i__0_ + *b*_i__1_t_ij_ + *ε*_ij_
Spline and random intercept	*NC*(*t*_ij_, 2, (*β*_0_, *β*_1_, *β*_2_, *β*_3_)^T^, (b_i__0_) +ε_ij_)
Spline and random slope	*NC*(*t*_ij_, 2, (*β*_0_, *β*_1_, *β*_2_, *β*_3_)^T^, (b_i__0_, b_i__1_)^T^ +ε_ij_)
Spline and random spline	*NC*(*t*_ij_, 2, (*β*_0_, *β*_1_, *β*_2_, *β*_3_)^T^, (b_i__0_, b_i__1_, b_i__2_, b_i__3_)^T^ +ε_ij_)

### Ethics Approval and Consent To Participate

Reuma.pt was approved by the National Data Protection Board (Comissão Nacional de Protecção de Dados, Portugal) and by the Ethics Committee of Centro Hospitalar Lisboa Norte, Hospital de Santa Maria, Lisbon, Portugal. Patients signed Reuma. pt's informed and written consent.

## Results

### Initial SpA Data Set

**Figure 4:**
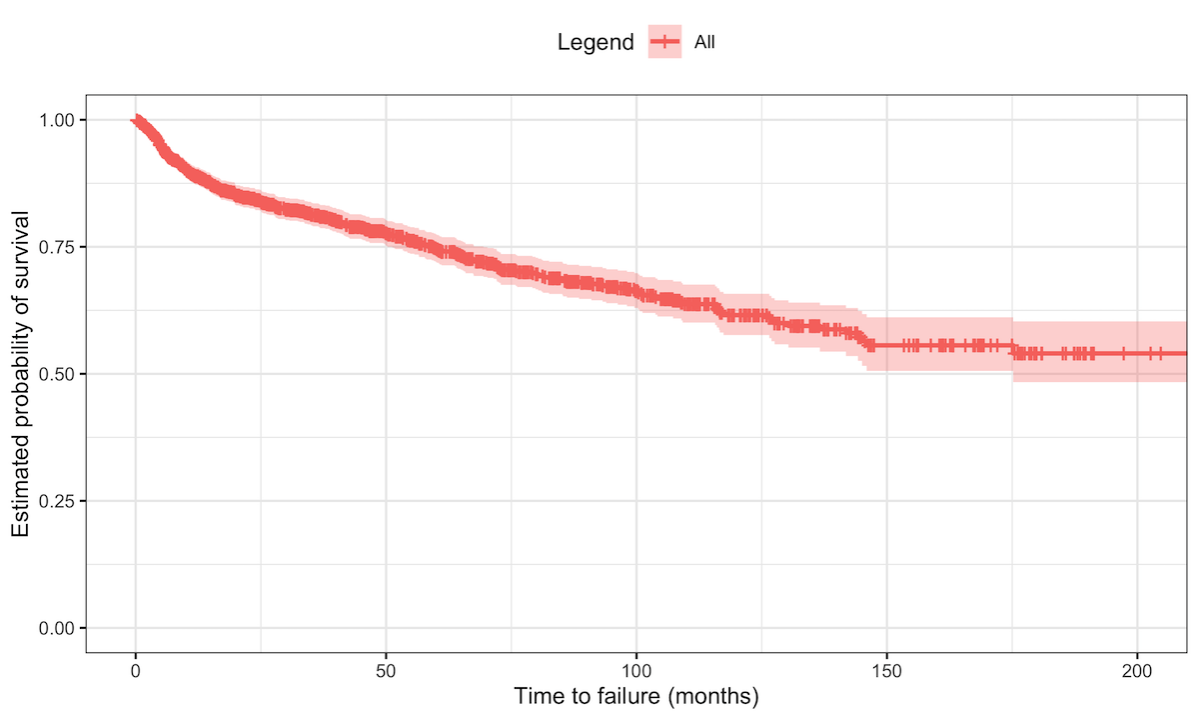
Kaplan-Meier estimation of the biologic therapy survival curve for the overall population of the initial data set with the 95% CI [5].

The survival probability curve, 
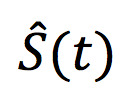
, of the first biologic therapy for the overall population from the initial data set obtained with the Kaplan-Meier estimator is presented in Figure 4, where vertical ticks along the curve indicate censored patients. We can observe that the slope of the curve is higher at the initial months, indicating that there are more failures closer to the beginning of the therapy.

**Figure 5:**
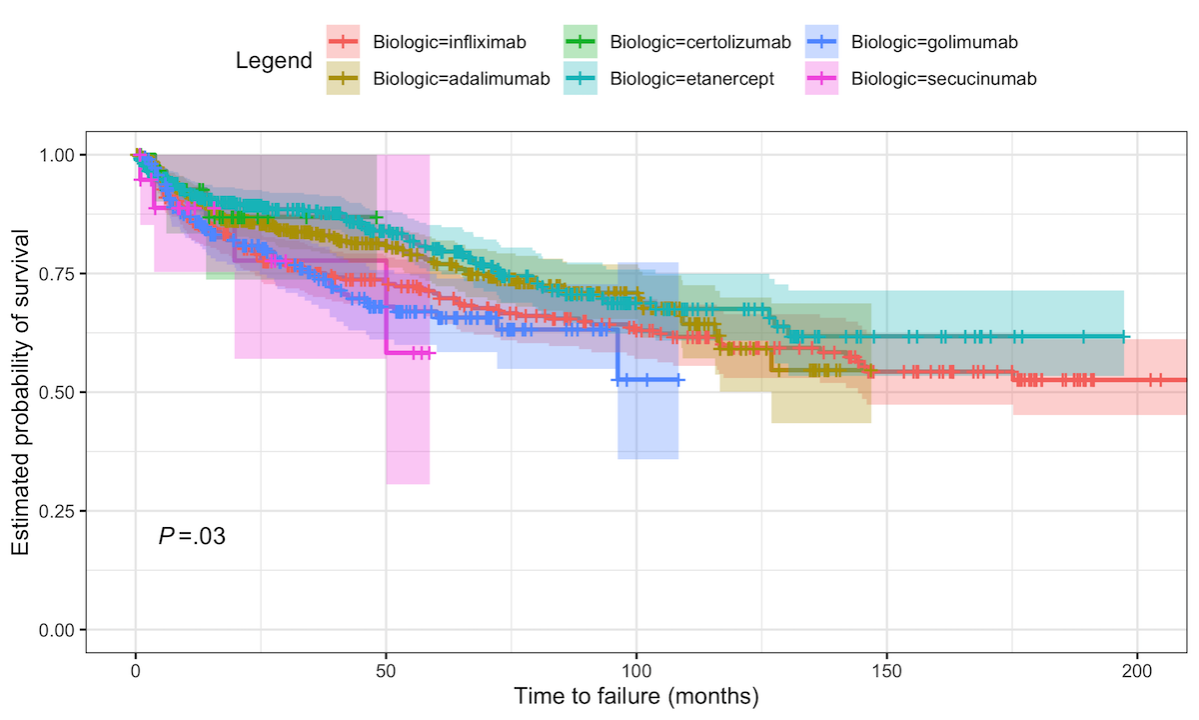
Kaplan-Meier estimation of the biologic therapy survival curve by biologic drug for the initial data set and *P* value of the correspondent log-rank test with the 95% CI [5].

A comparison of the survival probabilities between the different biologic therapies can be seen in Figure 5, where it is possible to observe a clear distinction between the different biologic drugs' curves. The *P* value of the log-rank test is also presented in the figure, indicating that the biologic drug curves differ significantly in survival at a 5% level.

A comparison of the survival probabilities between the different biologic therapies can be seen in Figure 5, where the survival curves for the different biologics were estimated using the Kaplan-Meier method. It is also possible to observe the *P* value of the log-rank test, whose null hypothesis is that all the groups have identical hazard functions. As this value is equal to .03, we can reject this hypothesis at a 5% level of significance.

The pairwise log-rank tests with corrections for multiple testing were also performed for all pairs of biologics to better compare the survival of the therapy between biologics. According to the tests, only 1 pair, etanercept and golimumab, had significantly different survival curves. An analysis of the curve indicates that golimumab conferred better survival than did etanercept at a 5% level of significance.

It should also be noted that in the Portuguese SpA subpopulation studied, there were some biologic drugs that had a very small number of observations. This difference in number of observations between different drugs could have also increased the difficulty in properly comparing the survival between them.

**Figure 6:**
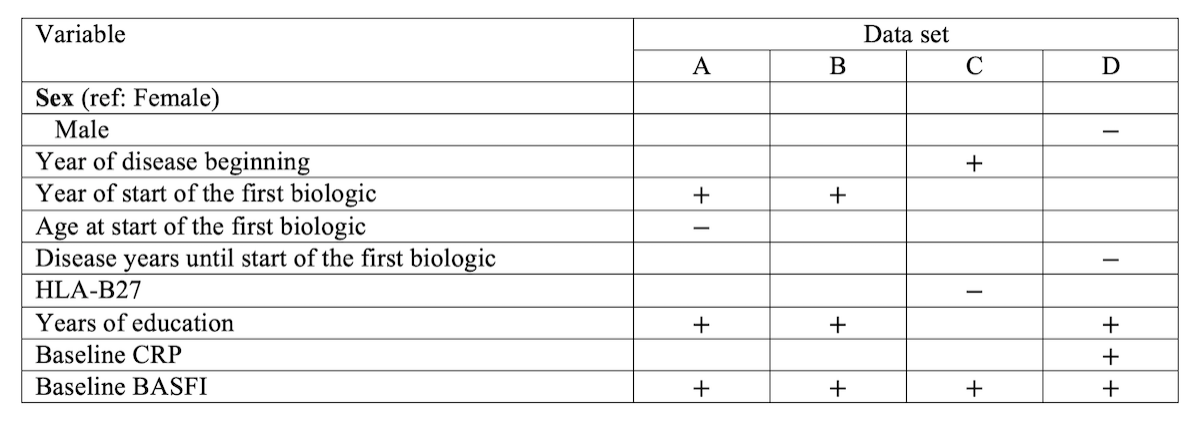
Coefficient signs of the covariates present in the survival submodels fitted with a Cox regression for data sets A, B, C, and D. BASFI: Bath Ankylosing Spondylitis Functional Index; CRP, C-reactive protein: HLA-B27, human leukocyte antigen B27.

### Comparison of Results: Data Sets A, B, C, and D

The first main step of the modeling process consisted of selecting the baseline variables of interest.

The comparison of the selected variables only took data sets A, B, and C into consideration. The percentage of times a variable was selected for each data set, considering the 5 variable selection approaches tested, is presented in Table 2, along with the average number of times it was selected overall.

Although not all variables were present in every data set, the variability in the covariates selected for each set of data was still noticeable. This indicates that the initial process of handling the missing values and the initial selection of variables to be considered at this stage have a somewhat elevated influence on the results that are obtained later; in short, the results are sensitive to the parameter choice. This difference may be justified by the existence of different variables and even different patients even if many are common between data sets.

Only 1 variable was selected by all methods and for all data sets where it was considered: years of education. On average, the variables that were selected in at least more than 50% of the methods used for variable selection were year of disease onset, age at start of the first biologic therapy, baseline BASFI, and baseline ASDAS.

Table 3 shows the sign of the coefficient for each of the covariates that were included in the survival submodel for each data set. The sign of the respective coefficient indicates the effect of this covariate on the outcome of interest, which we took as the failure of the first biologic therapy. A positive sign indicates that the variable increases the risk of failure for higher values of that variable (for a continuous variable) or for that value in comparison to the reference level (for a categorical variable); a negative coefficient indicates the opposite: a decrease in the risk of failure.

**Table 2:** Percentage of times a variable was selected for each data set (A, B, C, D) and the average of those percentages across all data sets.

Variable	Data set (%)				
	A	B	C	Average	
Sex	0	20	0	7	
Marital status	20	N/A^a^	N/A	20	
Year of diagnosis	20	40	20	27	
Age at diagnosis	40	40	N/A	40	
Year of disease onset	60	20	100	60	
Age at disease onset	20	20	N/A	20	
Year of start of the first biologic therapy	40	60	20	40	
Age at start of the first biologic therapy	60	40	N/A	50	
Years from diagnosis to start of the first biologic therapy	20	20	N/A	20	
Disease years until start of the first biologic therapy	20	20	N/A	20	
HLA-B27^b^	0	40	100	47	
Employment status before disease	40	20	N/A	30	
Employment status at baseline	20	20	0	13	
Years of education	100	100	N/A	100	
Smoking	40	40	20	33	
Alcohol	20	40	20	27	
Weight	40	N/A	N/A	40	
Height	20	N/A	20	20	
BMI	40	N/A	N/A	40	
Number of pathologies	20	0	N/A	10	
Biologic	20	20	20	20	
Concomitant DMARD^c^	0	20	0	7	
Concomitant corticoid	60	20	20	33	
Baseline CRP^d^	20	0	20	13	
Baseline ESR^e^	40	20	40	33	
Baseline BASDAI^f^	40	0	60	33	
Baseline BASFI^g^	80	100	40	73	
Baseline ASDAS^h^	60	40	60	53	

^a^N/A: not applicable.

^b^HLA-B27: human leukocyte antigen B27.

^c^DMARD: disease-modifying antirheumatic drug.

^d^CRP: C-reactive protein.

^e^ESR: erythrocyte sedimentation rate.

^f^BASDAI: Bath Ankylosing Spondylitis Disease Activity Index.

^g^BASFI: Bath Ankylosing Spondylitis Functional Index.

^h^ASDAS: Ankylosing Spondylitis Disease Activity Score.

**Table 3:** Coefficient signs of the covariates present in the survival submodels fitted with a Cox regression for data sets A, B, C, and D.

Variable		Data set			
		A	B	C	D
**Sex**					
	Female (ref)				
	Male				–
Year of disease beginning				+	
Year of start of the first biologic therapy		+	+		
Age at start of the first biologic therapy		–			
Disease years until start of the first biologic therapy					–
HLA-B27^a^				–	
Years of education		+	+		+
Baseline CRP^b^					+
Baseline BASFI^c^		+	+	+	+

^a^HLA-B27: human leukocyte antigen B27.

^b^CRP: C-reactive protein.

^c^BASFI: disease-modifying antirheumatic drug.

It can be noticed that the sign of the coefficient is coherent between the data sets for all variables even if the number of data sets where that variable is present differs.

According to the results obtained in the Cox regression, the factors that indicate a good prognosis for the biologic drug survival were being a male, starting the biologic therapy at an older age, having a larger time interval between disease start and initiation of the first biologic therapy, and being HLA-B27 positive. On the contrary, a disease onset or initiation of biologic therapy in more recent years, a higher number of years of education, and higher values of CRP or BASFI at baseline were all predictors of a greater risk of failure of the first biologic therapy.

Given the elevated number of models fitted and to aid in the drawing of comprehensive conclusions, Table 4 and Table 5 were created to depict the joint and extended Cox models, respectively. For each data set and for every variable present in the model, the tables show the percentage of models (relative to the total number of fitted models for each data set) in which the covariate was statistically significant, the percentage of models in which the variable had a positive regression coefficient, and the percentage of models in which the variable had a negative regression coefficient. Furthermore, the average of these percentages was calculated to obtain an overall view of the most common behavior of each variable as determined by information gathered from all data sets.

**Table 4:** Percentage of models in which a variable was statistically significant; percentage of models in which a variable had a positive coefficient sign; the percentage of models in which a variable had a negative coefficient sign for the covariates present in the joint models fitted for data sets A, B, C, and D; and the average of those percentages across all data sets.

Variable	Data set (%)																	Average (%)		
	A				B				C				D							
	ss^a^	pos^b^	neg^c^	ss		pos	neg	ss		pos	neg	ss		pos	neg	ss			pos	neg
Male (ref: female)	N/A^d^	N/A	N/A	N/A		N/A	N/A	N/A		N/A	N/A	0		46	54	0			46	54
Year of disease onset	N/A	N/A	N/A	N/A		N/A	N/A	92		75	25	N/A		N/A	N/A	92			75	25
Year biologic therapy initiation	82	36	64	83		67	25	N/A		N/A	N/A	N/A		N/A	N/A	83			52	45
Age at start of the first biologic therapy	82	0	100	N/A		N/A	N/A	N/A		N/A	N/A	N/A		N/A	N/A	82			0	100
Disease years until start of the first biologic therapy	N/A	N/A	N/A	N/A		N/A	N/A	N/A		N/A	N/A	15		0	100	15			0	100
HLA-B27^e^	N/A	N/A	N/A	N/A		N/A	N/A	83		0	100	N/A		N/A	N/A	83			0	100
Years of education	82	100	0	100		100	0	N/A		N/A	N/A	85		100	0	91			100	0
Baseline CRP^f^	N/A	N/A	N/A	N/A		N/A	N/A	N/A		N/A	N/A	44		100	0	44			100	0
Baseline BASFI^g^	50	100	0	75		100	0	25		63	37	50		100	0	50			91	9
CRP	67	100	0	100		100	0	100		100	0	100		100	0	92			100	0
ESR^h^	50	100	0	100		100	0	100		100	0	100		100	0	88			100	0
BASDAI^i^	100	100	0	100		100	0	100		100	0	100		100	0	100			100	0
BASFI	33	33	66	50		50	50	50		50	50	60		60	40	48			48	52
ASDAS^j^	100	100	0	100		100	0	100		100	0	100		100	0	100			100	0

^a^ss: percentage of models in which variable is statistically significant.

^b^pos: percentage of models in which variable has positive coefficient.

^c^neg: percentage of models in which variable has negative coefficient.

^d^N/A: not applicable.

^e^HLA-B27: human leukocyte antigen B27.

^f^CRP: C-reactive protein.

^g^BASFI: Bath Ankylosing Spondylitis Functional Index.

^h^ESR: erythrocyte sedimentation rate.

^i^BASDAI: Bath Ankylosing Spondylitis Disease Activity Index.

^j^ASDAS: Ankylosing Spondylitis Disease Activity Score.

**Table 5:** Percentage of models in which a variable was statistically significant; percentage of models in which a variable had a positive coefficient sign; and percentage of models in which a variable had a negative coefficient sign for the covariates present in the extended Cox models fitted for data sets A, B, C, and D; and average of those percentages across all data sets.

Variable	Data set															Average		
	A			B			C				D							
	ss^a^	pos^b^	neg^c^	ss	pos	neg	ss	pos	neg	ss		pos	neg	ss			pos	neg
Male (ref: female)	N/A^d^	N/A	N/A	N/A	N/A	N/A	N/A	N/A	N/A	0		25	75	0			25	75
Year of disease onset	N/A	N/A	N/A	N/A	N/A	N/A	100	100	0	N/A		N/A	N/A	100			100	0
Year of biologic therapy initiation	100	100	0	100	100	0	N/A	N/A	N/A	N/A		N/A	N/A	100			100	0
Age at start of the first biologic therapy	86	0	100	N/A	N/A	N/A	N/A	N/A	N/A	N/A		N/A	N/A	86			0	100
Disease years until start of the first biologic therapy	N/A	N/A	N/A	N/A	N/A	N/A	N/A	N/A	N/A	0		0	100	0			0	100
HLA-B27^e^	N/A	N/A	N/A	N/A	N/A	N/A	86	0	100	N/A		N/A	N/A	86			0	100
Years of education	100	100	0	100	100	0	N/A	N/A	N/A	75		100	0	92			100	0
Baseline CRP^f^	N/A	N/A	N/A	N/A	N/A	N/A	N/A	N/A	N/A	40		80	20	40			80	20
Baseline BASFI^g^	50	100	0	50	100	0	0	50	50	50		100	0	38			88	13
CRP	0	100	0	50	100	0	0	100	0	100		100	0	38			100	0
ESR^h^	0	100	0	100	100	0	100	100	0	100		100	0	75			100	0
BASDAI^i^	100	100	0	100	100	0	100	100	0	100		100	0	100			100	0
BASFI	33	100	0	67	100	0	33	100	0	100		100	0	58			100	0
ASDAS^j^	100	100	0	100	100	0	100	100	0	100		100	0	100			100	0

^a^ss: percentage of models in which variable is statistically significant.

^b^pos: percentage of models in which variable has positive coefficient.

^c^neg: percentage of models in which variable has negative coefficient.

^d^N/A: not applicable.

^e^HLA-B27: human leukocyte antigen B27.

^f^CRP: C-reactive protein.

^g^BASFI: Bath Ankylosing Spondylitis Functional Index.

^h^ESR: erythrocyte sedimentation rate.

^i^BASDAI: Bath Ankylosing Spondylitis Disease Activity Index.

^j^ASDAS: Ankylosing Spondylitis Disease Activity Score.

This consensus or ensemble approach was conducted to facilitate the identification of the most significant variables. The rationale is that if a feature always appears as significant, independently of the specific chosen model, then there is evidence that this feature is associated with the outcome.

Similar reasoning is applicable for identifying the covariates’ effect on the event of interest; that is, its positive or negative contribution for the risk of the therapy failure.

Focusing on the time-independent variables and starting with the covariate that represents male sex, we can see that this variable is not statistically significant in any joint or extended Cox model. This is coherent with what was observed in the Cox model, where sex was not a statistically significant predictor for our outcome. Regarding the effect of the variable on the event of interest, being male was more frequently a good predictor of biologic therapy survival than a bad predictor although this ratio was very small for the joint models.

The year of disease beginning was statistically significant for most models it was present in even if only 1 data set analyzed this variable. Its associated coefficient was positive for all the extended Cox models and for an average of 92% (12/13) of the joint models, which is consistent with the result obtained in the Cox models, indicating that patients with a more recent onset of disease have a higher risk of treatment failure.

The year of start of the first biologic therapy appears as a statistically significant predictor in most joint models and in all extended Cox models. For the majority of models, biologic therapy initiated in more recent years appeared to increase the risk of its failure.

The age at the start of the first biologic was statistically significant in most joint and extended Cox models, and older age at the time of therapy initiation was consistently a predictor of decreased risk of failure.

The year interval between disease beginning and start of the first biologic therapy was only statistically significant in a small percentage of joint models and showed no significance in any of the extended Cox models, echoing the results for the Cox model. The coefficient sign for this covariate was coherent among all models, indicating that a larger year interval reduces the chance of biologic therapy failure.

Being HLA-B27 positive was statistically significant as a predictor for biologic therapy failure in approximately 80% of all models and was consistently associated with a decreased risk of failure when in comparison with HLA-B27–negative patients.

The number of years of education showed statistical significance in roughly 90% of all joint and extended Cox models, and a higher number of education years increased the hazard of biologic therapy failure for all Cox, extended Cox, and joint models.

The value of CRP at baseline had an associated positive regression coefficient in all joint models and in 80% (4/5) of extended Cox models, indicating an increased risk of failure for higher CR*P* values, which was also verified in the Cox model. This covariate was not statistically significant in the Cox regression, but was significant in approximately 40% of the joint and extended Cox models.

The baseline BASFI was statistically significant in fewer than half of the joint and extended Cox models, even though it was always statistically significant in the survival submodels fitted with a Cox model. This variable appeared to be a predictor for increased risk of biologic therapy failure in most joint and extended Cox models, which is concordant with the Cox models' results.

Regarding the time-dependent variables, we noticed that, for the joint models, variables CRP, ESR, BASDAI, and ASDAS appeared to be statistically significant in most models. Furthermore, all were predictors of increased therapy failure for all the joint models that were fitted. Variable BASFI was only statistically significant in approximately half of the joint models, and the sign of its coefficient also varied considerably, not showing any clear tendency regarding the effect of this variable on the outcome.

In the extended Cox models, only variables BASDAI and ASDAS were statistically significant for all models. Variable ESR, BASFI, and CRP showed statistical significance in 75%, 58%, and 38% of the models, respectively. In all the models, all 5 time-varying covariates were predictors of increased risk of biologic failure.

## Discussion

### Principal Results

Overall, the results obtained from the Cox models, extended Cox models, and joint models all indicated similar effects of the covariates on the treatment outcome.

The biomarkers that indicated a good prognosis for the biologic drug survival were being male, starting biologic therapy at an older age, having a larger time interval between disease onset and initiation of the first biologic drug, and being HLA-B27 positive.

Conversely, disease onset or initiation of biologic therapy in more recent years, a greater number of education years, and higher values of CRP or BASFI at baseline all appeared to be predictors of a greater risk of failure of the first biologic therapy.

### Comparison With Prior Work

Male sex [22], HLA-B27–positive status [32], and longer disease duration [21] have been reported in the literature as being good predictors of biologic drug survival, which concurs with the results obtained in the Cox models of our study. On the other hand, older age at the start of the biologic therapy [32] has been reported to increase the risk of failure of the therapy, which is contrary to what was found in our data. We could interpret our result by speculating that older patients are more complacent due to the perceived efficacy of the therapy or because their symptoms are more intense than those of younger people and thus the relative improvement of symptoms is more noticeable, therefore increasing their satisfaction levels and decreasing the chances of therapy switch.

Regarding the predictors that were found to increase the risk of failure in our study, starting treatment in more recent years [33], and higher values of BASFI at baseline [21] were likewise found to be predictors of biologic drug discontinuation in research publications. A higher number of education years [23] was reported in one study as decreasing the risk of therapy failure, which differed from our results. Again, we could speculate and say that patients with a higher academic level are more comfortable with expressing their discontent with the lack of therapy response or that they are more aware of new therapeutic options and for that reason, request a switch of the therapy more often.

Higher values of CRP at baseline were found to increase [34] the hazard of biologic therapy failure in some publications but to have the opposite effect [23,33] in others.

### Limitations

Some limitations of our study include the suboptimal fitting of the longitudinal variables. Therefore, the choice of the LME function for describing the biomarker trajectories and the different functional forms available that specify the association between the longitudinal biomarker and the hazard function of the event should be further explored.

### Conclusions

Joint models are statistical models that can analyze both time-static and time-varying variables and therefore enable the inference of relationships between time-to-event and longitudinal data that are widely present in electronic medical records.

In this work, this modeling approach was selected to investigate biologic drug survival and its predictors for SpA patients in Portugal.

Furthermore, the insights obtained throughout the process that culminated in the fitting of these models are also highly valuable.

This study was the first to use the data of SpA patients from Reuma.pt in this capacity. The entire preprocessing work performed for enabling the use of Reuma.pt produced a data set that can be used by researchers who wish to investigate this group of diseases.

The variable selection process appears to be sensitive to this data preprocessing step depending on which variables and patients are described in the data set.

The tested methods for variable selection yielded quite different results for the same set of data. The process of selection of covariates should be analyzed carefully, as fully automated methods may not be the most appropriate ones for establishing which variables should be included in the statistical model. A wise approach consists of a balance between statistical significance and clinical significance, with the study's goal always being kept in mind.

We demonstrated that joint models, particularly the functions implemented in the R software packages “JM” and “Jmbayes,” can be successfully used for the simultaneous analysis of time-to-event and longitudinal data.

Health care providers use rheumatic disease progression measures computed from disease activity scores to shape treatment strategies and improve the quality of life of their patients [35]. However, targeted treatments that save patients from the potential side effects of high-cost, unsatisfactory treatments require the identification of biomarkers that can determine which patients can profit from a given therapy [36]. Computational methods using statistical and machine learning methods hold promise for the overall understanding of rheumatic diseases and can aid in formulating therapeutic strategies and predicting prognosis and outcome [37,38].

With this study, it was possible to identify the potential predictors of biologic therapy failure for this Portuguese population of SpA patients. This can aid the prognosis of these rheumatic diseases and potentially predict the most adequate treatment option according to the patient's characteristics.

## Abbreviations

AIC: Akaike information criterion
AS: ankylosing spondylitis
ASDAS: ankylosing spondylitis disease activity score
BASDAI: Bath Ankylosing Disease Status in Ankylosing Spondylitis
BASFI: Bath Ankylosing Spondylitis Functional Index
CRP: C-reactive protein
ESR: erythrocyte sedimentation rate
HLA: human leukocyte antigen
LME: linear mixed effects
SpA: spondyloarthritis
